# Data-driven modeling and control of an X-ray bimorph adaptive mirror

**DOI:** 10.1107/S1600577522011080

**Published:** 2023-01-01

**Authors:** Gautam Gunjala, Antoine Wojdyla, Kenneth A. Goldberg, Zhi Qiao, Xianbo Shi, Lahsen Assoufid, Laura Waller

**Affiliations:** a Department of Electrical Engineering and Computer Sciences, University of California, Berkeley, Berkeley, California, USA; b Advanced Light Source, Lawrence Berkeley National Laboratory, Berkeley, California, USA; c Advanced Photon Source, Argonne National Laboratory, Lemont, Illinois, USA; Australian Synchrotron, Australia

**Keywords:** adaptive optics, beamline optics, machine learning, control, X-rays

## Abstract

Adaptive X-ray mirrors are being adopted on high-coherent-flux synchrotron and X-ray free-electron laser beamlines where dynamic phase control and aberration compensation are necessary to preserve wavefront quality from source to sample, yet challenging to achieve. Additional difficulties arise from the inability to continuously probe the wavefront in this context, which demands methods of control that require little to no feedback. In this work, a data-driven approach to the control of adaptive X-ray optics with piezo-bimorph actuators is demonstrated. This approach approximates the non-linear system dynamics with a discrete-time model using random mirror shapes and interferometric measurements as training data. For mirrors of this type, prior states and voltage inputs affect the shape-change trajectory, and therefore must be included in the model. Without the need for assumed physical models of the mirror’s behavior, the generality of the neural network structure accommodates drift, creep and hysteresis, and enables a control algorithm that achieves shape control and stability below 2 nm RMS. Using a prototype mirror and *ex situ* metrology, it is shown that the accuracy of our trained model enables open-loop shape control across a diverse set of states and that the control algorithm achieves shape error magnitudes that fall within diffraction-limited performance.

## Abstract 2

A framework for data-driven characterization of the nonlinear dynamics of a piezo-bimorph adaptive X-ray mirror has been developed. Rapid surface shape control and stability to within 2 nm RMS have been demonstrated.

## 1. Introduction

The next generation of light sources, including free-electron lasers and diffraction-limited storage rings, will produce X-ray beams of unprecedented brightness and coherent flux, enabling fast experiments where wavefront phase information will be used to study matter in exquisite detail.

Reflective X-ray optics (*e.g.* mirrors and gratings) are illuminated at glancing angles of incidence and their surface shape tolerances are on the scale of nanometres. Achieving high Strehl ratios from beamlines with several mirrors requires that individual-mirror height errors be limited to the nanometre scale, depending on the wavelength (Shi *et al.*, 2016 ▸). To reach efficient diffraction-limited X-ray optical performance in routine operation, it is necessary to correct residual aberrations arising from imperfect optical surfaces, misalignment, thermo-mechanical deformations and dynamic mirror shape deformations caused by time-varying power loads and beam profiles (Sanchez del Rio *et al.*, 2020 ▸; Cutler *et al.*, 2020 ▸).

The development of X-ray adaptive optics started in the mid-1990s (Susini *et al.*, 1996 ▸). Over the last decade, significant advances (Mimura *et al.*, 2010 ▸; Sawhney *et al.*, 2010 ▸) have led to the commercial availability of piezo-bimorph mirrors (Alcock *et al.*, 2019*a* ▸; Ichii *et al.*, 2019 ▸) and their successful deployment on several beamlines (Matsuyama *et al.*, 2016 ▸; Sutter *et al.*, 2019 ▸). A recent review by Cocco *et al.* (2022 ▸) summarizes alternative approaches to deformable mirrors. These mirrors have symmetrically placed bimorph elements attached to silicon mirror substrates, which allow these systems to maintain thermal stability while providing one-dimensional shape actuation. Investigations of the mirrors’ linear response demonstrate that their shape along the tangential (longitudinal) direction can be controlled to a nanometre level in a predictive way (Vannoni *et al.*, 2016 ▸; Alcock *et al.*, 2019*a* ▸) as required for diffraction-limited performance. Studies of the mirrors’ dynamic response show that appreciable shape changes on the scale of 1 s are possible (Alcock *et al.*, 2019*b* ▸), as well as precise actuation relying on closed-loop feedback with accuracy better than 1 nm using arrays of laser interferometers (Alcock *et al.*, 2019*c* ▸).

Measuring the performance of one such piezo-bimorph mirror using X-ray light and a wavefront sensor, we have observed time-dependent and history-dependent behaviors that defy a simple linear response model. The use of piezo-electric materials to induce deformation of relatively thick substrates is associated with nonlinearities such as cross-talk between actuators, creep and hysteresis (Alcock *et al.*, 2015 ▸). The nanometre-scale magnitudes of these effects are relevant for our applications. Our work confronts the challenge of controlling the mirror shape in the presence of dynamic non-linear behavior. For soft X-ray applications, in particular, wavefront monitoring interrupts the beam delivery. With the increased ability to predict the temporal behavior following actuation, fewer wavefront measurements are required to achieve and maintain the desired shape, and systems progress toward the goal of open loop operation.

Current approaches for *in situ* mirror shape control rely on a linear model. Nonlinearities are usually compensated using closed-loop feedback from an X-ray wavefront sensor (Assoufid *et al.*, 2016 ▸; Liu *et al.*, 2018 ▸; de La Rochefoucauld *et al.*, 2018 ▸; Goldberg *et al.*, 2021 ▸; Shi *et al.*, 2020 ▸) or *in situ* monitoring (Badami *et al.*, 2019 ▸). In practice, beam pick-up for feedback can be invasive or interrupting, and systems that can operate in open-loop are desirable. Moreover, while linear models may be adequate for small surface changes, they have significant limitations for larger moves and fail to capture the dynamic response at short (seconds) and long (minutes) time scales. Comprehensive physical modeling of the mirror (*e.g.* using finite-element analysis) is possible (Song *et al.*, 2009 ▸; Jiang *et al.*, 2019 ▸), but it requires highly specific system characterization, which cannot always be achieved in practice. In recent years, similar complex systems such as the storage ring itself are now using techniques derived from machine learning to improve their stability (Leemann *et al.*, 2019 ▸). Given their success, we aim to apply similar data-driven techniques to the operation of adaptive X-ray optics and circumvent the limitations of linear methods.

## 2. Methods

In this paper we propose a two-part framework for the open-loop operation of an X-ray deformable mirror, involving (1) approximating the nonlinear system dynamics using a feedforward neural network, and (2) control to a desired surface shape using nonlinear quadratic cost regulation over a finite time horizon. We developed and tested our approach using an *ex situ* visible-light Fizeau interferometer to record the behavior of an adaptive mirror driven through various shape transitions. The test mirror is a PZT (lead zirconate titanate)-glued bimorph mirror fabricated by JTEC Corporation (see Appendix *A*.1[App appa] for details). Our methodology is broadly applicable to X-ray or other optical systems utilizing an adaptive element, independent of the optical configurations and wavelength ranges.

### 2.1. Predictive modeling

**Figure 1:**
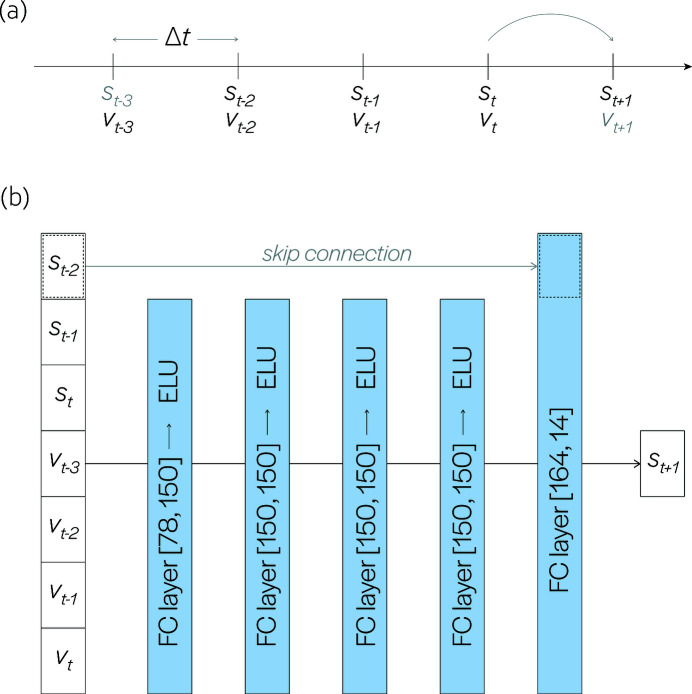
Discretization and neural network architecture. (*a*) Our discrete-time model aims to predict the shape of the mirror at a time Δ*t* in the future (*s*_*t*+1_) using a finite history of mirror shapes and voltages input to actuators. Note that *s*_*t*−3_ and *v*_*t*+1_ are not used in the prediction of *s*_*t*+1_. (*b*) Our learned system dynamics model consists of five fully connected (FC) layers ([input dimension, output dimension]) followed by exponential linear unit (ELU) activation functions. Additionally, a skip connection was introduced which greatly improved its ability to predict when the mirror was at or close to rest.

We want to find a discrete time model for the nonlinear dynamics of the bimorph mirror with the general form 

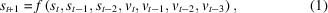

where *s*_*t*_ and *v*_*t*_ represent the mirror surface and voltage input applied to each actuator at discrete times *t*, respectively. For a fixed time step of Δ*t*, the model should predict the shape of the mirror one time step in the future given its current shape, current input voltage, and a finite history of shapes and inputs [see Fig. 1 ▸(*a*)]. The number of past states and inputs was chosen empirically based on observed performance, and our time step of Δ*t* – 2.0 s was limited by interface latency with the prototype mirror.

We use a feedforward neural network (Bishop, 2006 ▸) as our discrete-time forward model for the nonlinear dynamics of the bimorph mirror, with five fully connected layers and exponential linear unit activation. An additional skip (or shortcut) connection was introduced due to their effectiveness in modeling an identity mapping (Bishop, 2006 ▸; He *et al.*, 2015 ▸); in our case, this greatly improved the predictive performance when the mirror was at or close to rest. The network architecture is shown in Fig. 1 ▸(*b*). The size of the input layer is determined by the dimension of our surface representation, the number of actuators being controlled and the amount of history incorporated in making a prediction. Due to the limited field-of-view (FOV) of the Fizeau interferometer being used to measure the mirror surface, we restrict actuation and analysis to the central 9 of 18 actuators. The mirror surface within the FOV is parametrized by heights at 14 equidistant points. A history of three discrete-time inputs and measurements, in addition to the input at the current time, are concatenated and treated as the input to a neural network that predicts the surface shape at the next time step.

To train our model, we collect a large ensemble of surface profiles occurring with sequences of random applied voltage inputs. The application of inputs and measurements of the mirror surface are synchronized according to our discrete-time model. In this study, the voltage input to an individual actuator is limited to a range of [−100 V, 100 V].

**Figure 2:**
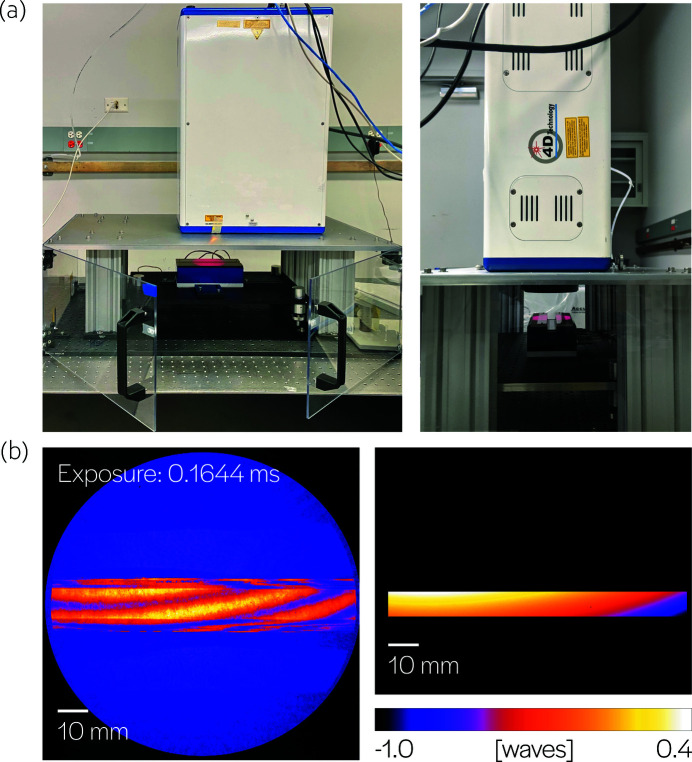
Experimental setup. (*a*) Mirror surface profiling with a Fizeau interferometer (λ = 658 nm) mounted vertically above the bimorph mirror. (*b*) An example interferogram. Four such measurements are acquired simultaneously and used to recover a surface profile, which is cropped to the active area of the mirror.

The surface measurements are acquired using a 4D Technology FizCam Fizeau interferometer, shown in Fig. 2 ▸. Appendix A.1[App appa] contains more details about the setup. To minimize the contribution of small vibrations, piston and tilt terms are removed from the 2D surface measurements. The surface is averaged in the narrow sagittal direction to produce 1D curves along the tangential dimension of actuation. The sequential data are divided into sub-sequences which constitute input–output training examples for the supervised learning of our dynamics model. A total of 5964 examples were collected and used to train the neural network. A detailed description of the data acquisition and learning process can be found in Appendix A.2[App appa].

### 2.2. Control

Once the parameters of the nonlinear system dynamics model, *f*(·), are learned, the model is used to determine a sequence of voltage inputs that will drive the mirror from a measured initial state to a desired final state in a given finite number of steps. Our algorithm solves a quadratic cost function, similar to the iterative linear quadratic regulator (Li & Todorov, 2004 ▸), that penalizes state error at each simulated intermediate step. However, rather than linearizing the system dynamics around the initial state and finding an analytic solution to the reduced problem, we directly minimize the non-convex objective function using the Adam algorithm (Kingma & Ba, 2014 ▸). While this does not theoretically guarantee optimality of the converged solution, the observed performance meets our specifications in a majority of experiments.

The cost function is given by

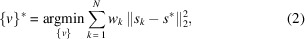

subject to 

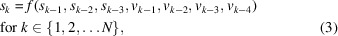

with initial conditions 

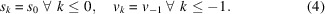

In this equation, *N* is the total number of computed steps, {*v*} represents the set of inputs {*v*_0_, *v*_1_,…*v*_*N*−1_}, 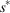 is the desired mirror shape, and the *w*_*k*_ coefficients apply a weighted penalty to the shape error at each time step. For the experiments discussed in this paper, we assume the first input, *v*_0_, is applied at time step 0, and that the mirror begins at rest with some arbitrary shape. Additionally, we use constant weights *w*_*k*_ in these experiments.

## 3. Results and discussion

To test the predictive performance of our learned system dynamics model, we collect a sequential test dataset similar to our training data. The voltages applied to the mirror actuators are updated and measurements of the surface are acquired at fixed time intervals (2, 12, 30 s). The learned model is applied to sub-sequences of these data, and the prediction is compared with the next measurement. Our test dataset consists of a total of 497 input–output examples. Here we evaluate the performance of our model when used with different time intervals, and in comparison with a linear-response model.

### 3.1. Comparison with a linear-response model

We compare the performance of our model with that of a linear prediction based on ‘influence functions’ (also called ‘actuator response functions’ or ‘characteristic functions’) (Hignette *et al.*, 1997 ▸; Goldberg & Yashchuk, 2016 ▸; Merthe *et al.*, 2012 ▸). In that approach, voltage is supplied to individual actuators in isolation and the resulting surface is measured once the mirror has settled. The set of shape measurements is treated as a basis, and a linear model predicts the resultant shape from an arbitrary set of inputs. To achieve a desired shape, the actuation matrix is then inverted using least-squares in order to obtain the corresponding input voltages. In practice, a linear model such as this is applied iteratively, with measurement at each step, to overcome discrepancies with the real-world response and converge to the target shape in several steps; this is known as feedback control.

**Figure 3:**
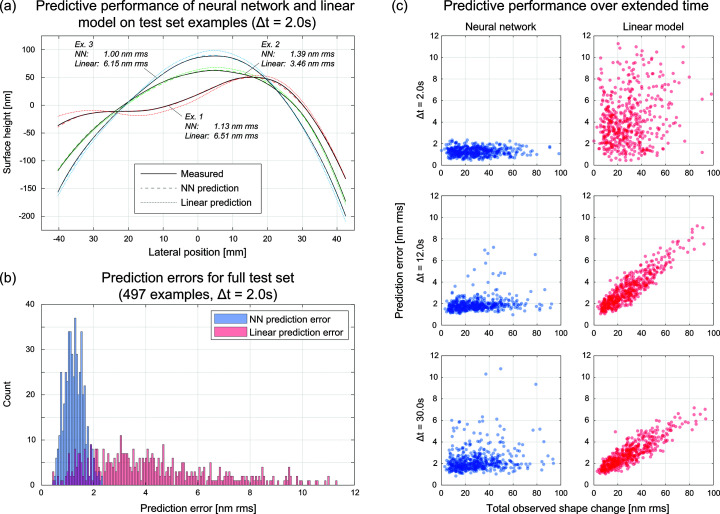
Performance of predictive model on test data. (*a*) Predicted curves from the neural network and linear models for three examples in the test dataset. (*b*) Predictive performance of neural network and linear models across a full test dataset. (*c*) Prediction errors of neural network and linear models plotted against the observed RMS change in surface shape for three different time scales. For time scales larger than 2 s, the neural network model is iteratively applied.

Some examples of shape prediction are demonstrated in Fig. 3 ▸(*a*), and the corresponding prediction errors for neural network (our method) and linear prediction are labeled. The aggregate performance over the entire test dataset is shown in Fig. 3 ▸(*b*). Overall, the mean prediction error for our method was 1.26 nm root mean square (RMS), compared with 4.20 nm RMS for linear prediction. Our neural network model demonstrated lower prediction error than the linear model in 460/497 test examples. The cases for which linear prediction demonstrated lower prediction error generally involved very small changes in input voltage.

It is well known that the linear model defined as a basis of influence functions is more appropriate for small changes in surface shape than for large changes. Moreover, since the influence functions are measured after waiting for the mirror to settle, they fail to capture dynamics over small time scales. In Fig. 3 ▸(*c*), the prediction errors for both models are plotted against the magnitude of observed shape change for a variety of time scales.

### 3.2. Varying the time interval

While the training data are acquired using a fixed 2 s time interval, we can apply the forward prediction process to a variety of time intervals, and compare with linear modeling. For all time intervals tested, the linear prediction is computed using a basis of influence functions measured at steady state. Testing the model’s predicted system response with a 2 s time interval gives the highest measured accuracy because the test data are acquired through a procedure identical to that of the training data. At this short time scale, errors from the linear model are essentially random since transient effects are poorly characterized by influence functions measured at steady state.

For longer time scales, we apply our learned model iteratively, using predicted intermediate surface shapes as input for subsequent steps toward the goal shape. For 12 s intervals (six prediction steps), we see that the neural network prediction performs worse on a somewhat sparse set of examples, and linear prediction begins to exhibit a linear correlation between the shape change observed (requested) and error. For 30 s intervals (15 prediction steps), neural network predictive performance degrades over a larger set of examples, and linear prediction maintains its linear correlation, albeit with lesser slope.

Unsurprisingly, the tests show that the predictive performance of our neural network model is best at the time interval used to acquire the training data; its performance degrades when used at longer time intervals. This is likely due to the much more limited representation of repeated inputs in our training data set. As shown in Appendix A.2[App appa], the training data set extracted from measurements with repeated inputs (no voltage input change) is only one-third of the total training data set. Also, the maximum number of consecutive measurements with repeating voltage inputs is only five, corresponding to a maximum of 10 s mirror relaxation without varying voltages. While the network effectively predicts shape changes when all voltage inputs are updated, more data are required to inform the network of convergence behaviors at different states when the inputs are held constant. We believe this can be improved with the acquisition of more finely sampled data. The results also demonstrate that linear-model prediction can be effective for small shape changes (<20 nm RMS) over long time scales. However, linear prediction, without repeated measurement and iteration, may be an inappropriate choice for applications where speed and open-loop operation are important.

### 3.3. Directed shape control

In practice, mirror shape control will be used to compensate phase errors in the wavefront of a focused beam. Therefore, it is of central importance to be able to direct the mirror to achieve and hold arbitrary shapes within its capabilities.

As a demonstration, we test the ability of our control algorithm to direct the mirror to a series of 50 random prescribed shapes. For each target shape, an initial surface measurement is acquired and used to generate a ten-step sequence of voltage inputs, to achieve the shape and stabilize the mirror. We allow 150 s to elapse between experiments so that the initial conditions [equation (4)] are approximately true.

**Figure 4:**
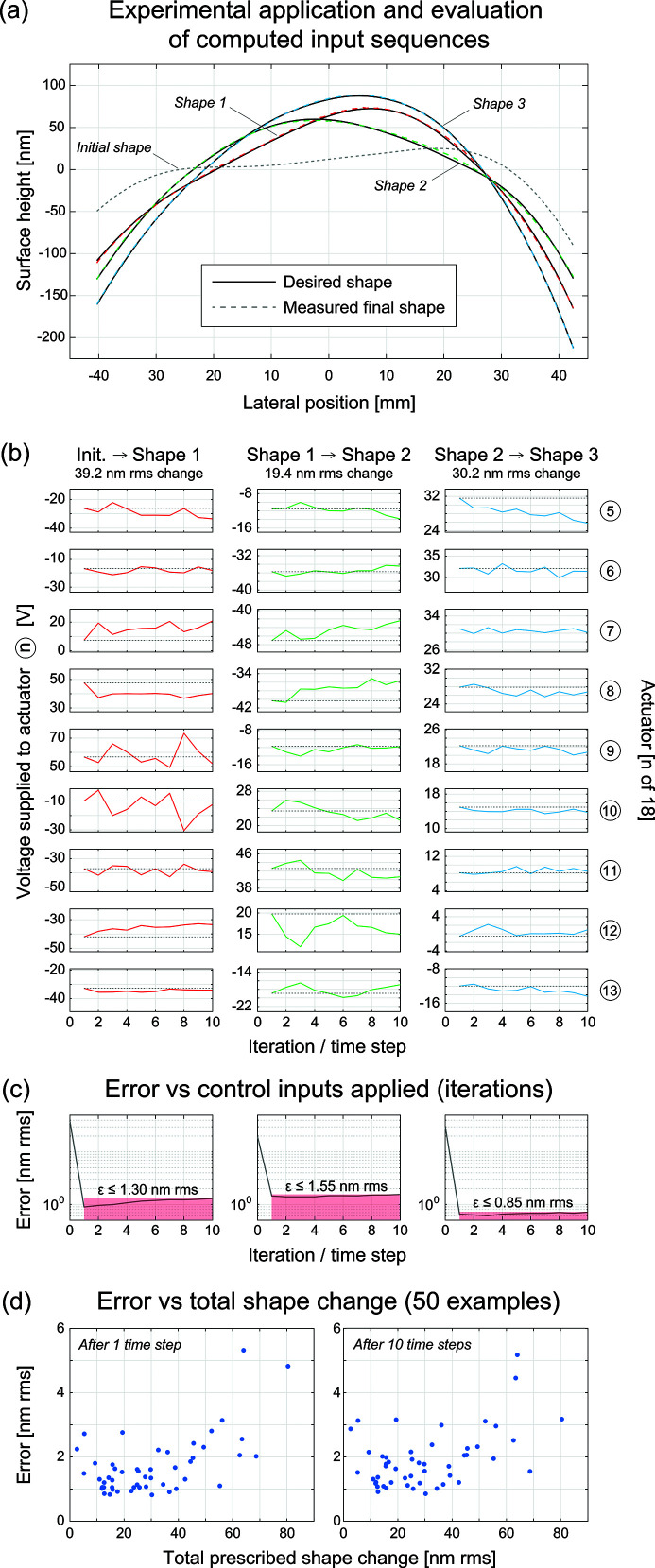
Controlling the mirror to desired surface shapes. (*a*) Measurements of the mirror surface taken over a sequence of three prescribed shapes. (*b*) Voltages applied to actuators over ten solved time steps for the three shape transitions. (*c*) Total shape error in nm RMS over ten time steps. (*d*) Aggregate performance of the control algorithm for the full test set of 50 prescribed random shapes.

The results of these experiments are shown in Fig. 4 ▸. A selected sequence of transitions to three prescribed shapes is shown in Fig. 4 ▸(*a*), where the measured surface profiles (colored, dashed) closely approximate the desired shapes (black, solid). Figure 4 ▸(*b*) shows the voltages applied to each of the nine actuators over the ten steps that were generated by the optimization algorithm.

We observe that these voltage sequences sometimes demonstrate oscillatory behavior, suggesting that the model is accommodating dynamic effects such as overshoot and creep. In Fig. 4 ▸(*c*), we see that the algorithm drives the mirror close to the goal after the first step, with the remaining nine steps being used to maintain the position. Some overshoot may still occur, as the error with respect to the prescribed shape is often slightly larger after ten steps than after the first step. This may be caused by a combination of the non-convexity of the optimization problem and the lack of guaranteed optimality, and by any residual errors in the predictive capability of our learned system dynamics model. The former can be somewhat addressed by changing the parameters of the Adam algorithm (learning rate, iterations) or the weights *w*_*k*_ in equation (2), or by adding regularization to the objective function, *e.g.* penalizing RMS differences between time-adjacent voltage inputs or predictions (‘velocity’). Figure 4 ▸(*d*) shows the aggregate performance of our control algorithm across the 50 test cases. The mean RMS errors between the measured and prescribed shapes are 1.70 nm after the first step and 1.91 nm after ten steps.

**Figure 5:**
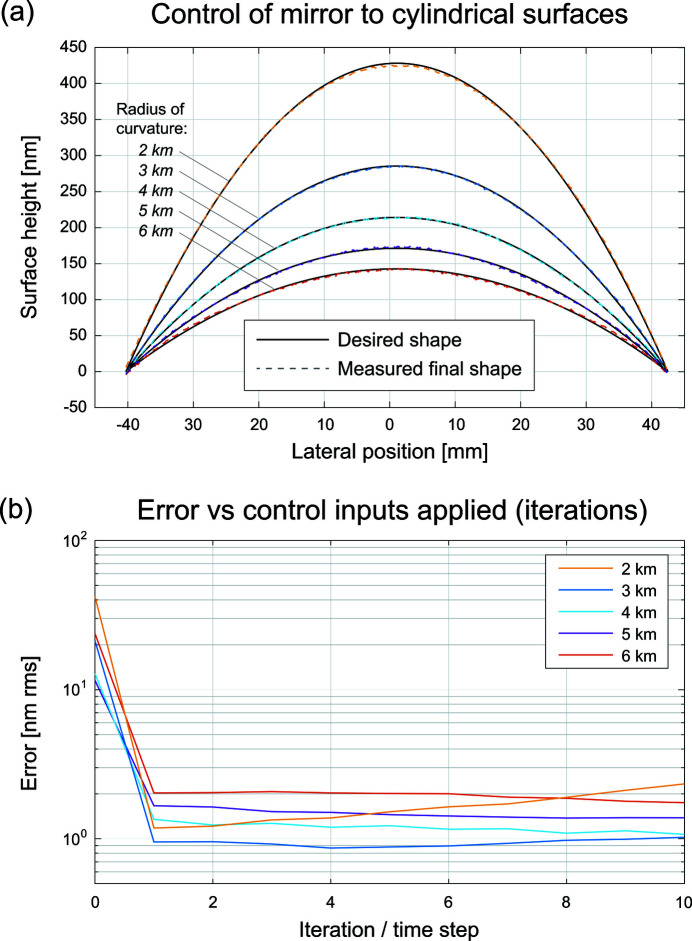
Controlling the mirror to cylindrical surface shapes. (*a*) Measurements of the mirror surface taken after the ten-step control algorithm is used to achieve cylindrical surface shapes. The piston was added to desired and measures surface profiles for visualization only. (*b*) Total shape error in nm RMS over ten time steps.

Among the directed shape-control tests, we drove the mirror to a set of cylindrical shapes with prescribed radii of curvature from 2 km to 6 km. This emulates the case of an adaptive mirror used to vary the focal distance, as in Sutter *et al.* (2019 ▸). In our applications, we consider these to be relatively large moves, with central surface height changes from 146.3 nm in the 6 km case to 429.0 nm in the 2 km case. Test results are shown in Fig. 5 ▸. We observe that the mean RMS errors between the measured and prescribed shapes are 1.44 nm after one step and 1.51 nm after ten steps.

## 4. Conclusion

We have shown that the combination of a data-driven model for piezo-bimorph adaptive mirror shape dynamics and an optimization-based control strategy was able to reduce residual mirror figure errors in open-loop operation below 2 nm RMS, outperforming linear models and achieving the shape-control accuracy required to achieve diffraction-limited performance in the X-ray regime.

Our method effectively accounts for creep and hysteresis, nonlinear properties that currently limit the performance of such devices in open-loop operation. Accurate predictive modeling to achieve stable arbitrary surface shapes is essential for effective deployment on high-coherent-flux X-ray beamlines where continuous feedback may be difficult to implement.

This calibration method is simple to implement and easily automated, requiring only a sequence of random shape commands and surface profile measurements. The data can be collected *ex situ*, as presented in this paper, or even *in situ* with a wavefront sensor, where the phase of the beam can be mapped back into the mirror shape if required. The method is also robust, providing accurate predictions and control across the full range of operation of the mirror. Other types of adaptive mirrors, such as resistive-element mirrors (Cocco *et al.*, 2020 ▸), can also be characterized with this technique.

The number of shape measurements required to build the training dataset is larger than what is required to acquire the characteristic functions in the linear model, but the training data can be gathered during routine operation, over time. There is some flexiblity around the structure of the neural network itself (hyperparameters such as number of inputs, layers), but the performance level we found is very close to the noise level of our sensor, and needed no further refinement despite being rather economical.

## Figures and Tables

**Figure 6:**
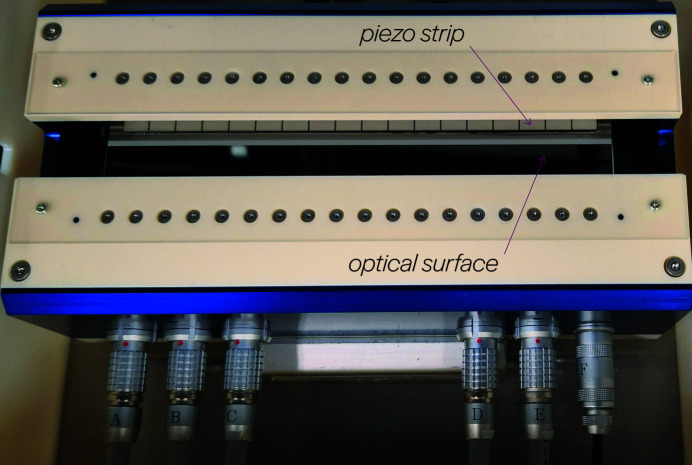
Piezo-bimorph deformable X-ray mirror. The adaptive X-ray mirror (JTEC Corporation) that was studied and characterized in our experiments. A segmented piezo strip enables spatially localized deformations of the optical surface.

## Appendix A

### A1. Experimental setup

The bimorph mirror was fabricated by JTEC and measured with a metrology setup designed in-house at the Advanced Photon Source, using a FizCam 2000 Fizeau interferometer (4D Technology) with 100 mm aperture. The interferometer is oriented vertically to measure mirrors with the optical surface facing upwards. The setup includes a manual tip-tilt stage for high-precision mirror alignment. A Lexan enclosure is constructed to enclose the X-ray mirror and the transmission flat of the interferometer to avoid air turbulence and reduce temperature fluctuation.

The bimorph mirror substrate (enclosed in the mirror box as shown in Fig. 6 ▸) has an overall dimension of 160 mm (length) × 50 mm (width) × 10 mm (thickness). The Pt-coated optical surface is 150 mm (length) × 8 mm (width), centered on the top side of the mirror. There are two piezo strips (one strip is visible in Fig. 6 ▸), each with 18 separate electrodes, that sandwich the optical surface. The two electrodes at a corresponding position of the two strips comprise one electrode channel (referred to as the actuator in this paper), which locally perturbs the optical surface. There are a total of 18 such channels (CH1 to CH18). There are two additional piezo strips glued to the bottom surface of the mirror, which act as a single electrode channel (CH20) that globally perturbs the optical surface. The grounded channel (CH19) is on the back side of all piezo strips and adjacent to the mirror surface. The resting state of the mirror is defined as when all 20 channels are at the same voltage. In this work, we kept CH19 and CH20 at the fixed voltage of 500 V and only varied the top-surface channels in a relative range of [−100 V, 100 V] around their nominal value of 500 V. Since the Fizeau interferometer aperture (100 mm) is smaller than the mirror length, we only used the nine central channels (CH5 to CH13).

### A2. Data acquisition and learning process

The data used to train our neural network model were acquired by applying voltages to the central nine mirror actuators and recording the resulting mirror shapes after a fixed, 2 s, time interval. An acquisition consists of ten images captured with an exposure time of 0.126 ms and averaged together. Acquisitions are triggered such that this exposure time does not contribute delays to the 2 s interval. The voltages are uniform random values in the range [−100 V, 100 V] and are rounded to the nearest tenth of a volt. A time interval of 2 s between the application of voltage inputs and surface measurement is maintained throughout the acquisition of training data.

We acquire eight separate sequences of surface measurements, starting with an initial measurement followed by 500 voltage changes. We acquire an additional four sequences of 501 measurements in which voltage changes are held for five time steps (*i.e.* the same voltage input is applied for five consecutive measurements). We believe these data are vital for informing the model of system convergence behavior. Since each training example requires four sequential measurements (one state to be predicted from three inputs), we can extract a maximum of 497 training examples from each of the 501-measurement sequences. In total, the 12 sequences give 5964 training examples.

We used the PyTorch library as the machine learning framework. To train our dynamics model, we perform a 15-fold cross validation on the training dataset with 3000 iterations of the Adam algorithm per fold. To test the model’s performance, we acquire another independent sequence of measurements and divide into sub-sequences, as described above, resulting in 497 test examples.
